# Engineered hollow Prussian blue nanoparticles for synergistic anti-inflammatory therapy in sepsis

**DOI:** 10.1016/j.mtbio.2026.103619

**Published:** 2026-08-28

**Authors:** Yang Song, Xue Yu, Mingzhe Wu, Jing Liu, Zhifeng Wen, Daosong Dong, Fangdie Ye, Shijie Zhang

**Affiliations:** a Department of Anesthesiology, Shengjing Hospital of China Medical University, Shenyang, China; b Department of Surgical Oncology and General Surgery, The First Hospital of China Medical University, Shenyang, Liaoning, China; c Department of Gynecology, The First Affiliated Hospital of China Medical University, Shenyang, China; d Department of Obstetrics, The First Hospital of China Medical University, Shenyang, China; e Department of Neurosurgery, The First Hospital of China Medical University, Shenyang, Liaoning Province, 110001, China; f Department of Pain, The First Hospital of China Medical University, Shenyang, Liaoning Province, 110001, China; g Fudan Institute of Urology, Huashan Hospital, Fudan University, Shanghai, China

**Keywords:** Sepsis, cfDNA, Nanoparticles, Hollow Prussian blue, Lipopolysaccharides

## Abstract

Sepsis is a systemic inflammatory response triggered by pathogenic infections, often accompanied by bacterial endotoxin release, accumulation of cell-free DNA (cfDNA), and excessive inflammatory reactions, leading to organ failure. Conventional antibiotic therapies are often inadequate in effectively eliminating pathogens and their toxic components, and fail to suppress the cfDNA-mediated inflammatory cascade. In this study, we developed a multifunctional nanotherapeutic platform based on hollow Prussian blue nanoparticles (HPB-NPs) loaded with polymyxin E (PME) and surface-modified with polyethylenimine (PEI). This versatile nanosystem (HPB-NPs@PME@PEI) exhibits potent antimicrobial activity against Gram-negative bacteria and specifically neutralizes lipopolysaccharides (LPS); PEI captures elevated levels of LPS and cfDNA in septic blood via electrostatic adsorption, thereby blocking its activation of the TLR4/MyD88 pathway and mitigating the cytokine storm; meanwhile, HPB scavenges reactive oxygen species (ROS), alleviating oxidative stress damage. Experimental results demonstrate that HPB-NPs@PME@PEI significantly reduces plasma cfDNA levels, suppresses the release of pro-inflammatory cytokines, and improves survival rates in a murine sepsis model. HPB-NPs@PME@PEI directly suppress LPS-induced inflammatory activation and NETosis in innate immune cells. Furthermore, by scavenging ROS and blocking the TLR4/MyD88/NF-κB pathway, HPB-NPs@PME@PEI effectively protects lung and renal epithelial cells from LPS injury. Thus, the nano-system operates via a synergistic mechanism, concurrently neutralizing LPS, clearing ROS, and inhibiting a major inflammatory signaling cascade.

## Highlights

• This study engineers a hollow Prussian blue nanoparticle loaded with polymyxin E and coated with PEI for sepsis therapy.
• The nanoplatform simultaneously scavenges ROS, neutralizes LPS/cfDNA, and inhibits the TLR4/MyD88/NF-κB pathway.
• In a murine sepsis model, the therapy improves survival and alleviates multi-organ (lung/kidney) injury.

## Graphical abstract

**Figure:**
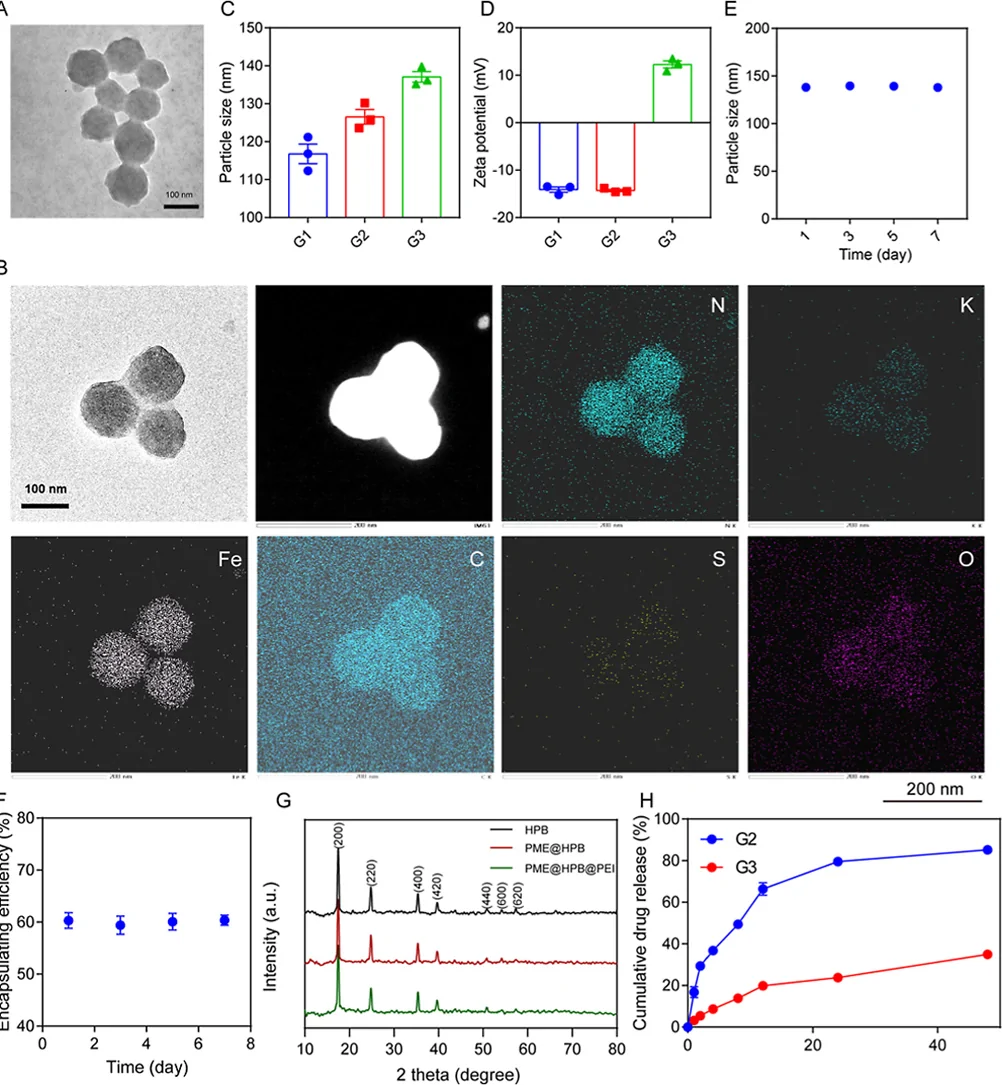

## 1 Introduction

Sepsis is defined as life-threatening organ dysfunction caused by a dysregulated host response to infection [1,2]. With its high global incidence and rapid progression, it remains a major challenge in critical care medicine [3]. The pathophysiology of this condition extends far beyond the direct damage inflicted by pathogens, such as bacteria or fungi. Rather, the core of the disease lies in the exaggerated activation of the immune system, leading to a “cascade” of inflammatory responses [4]. This process is driven by two major classes of signaling molecules: pathogen-associated molecular patterns, such as lipopolysaccharide released by Gram-negative bacteria, and damage-associated molecular patterns released by injured host cells [5]. Among the latter, cell-free DNA—generated via neutrophil extracellular traps or other mechanisms—has been identified in recent years as a key player [6]. These molecules collectively activate innate immune signaling pathways, triggering a burst release of pro-inflammatory cytokines including TNF-α and IL-6[[7], [8], [9]]. This results in a “cytokine storm,” accompanied by severe oxidative stress, ultimately leading to disseminated intravascular coagulation, circulatory failure, and multiple organ failure. Although broad-spectrum antibiotics remain the first-line clinical standard, their mechanism of action is limited[[10], [11], [12], [13]]. While they may clear certain pathogens, they neither effectively neutralize persistent toxins, such as endotoxins nor interrupt the sustained inflammatory state mediated by endogenous alarm signals like cfDNA [14]. Moreover, antibiotic-induced bacterial lysis may even exacerbate toxin release, which helps explain why sepsis mortality remains persistently high[[15], [16], [17], [18]]. In periodontitis, inflamed gums with compromised epithelial barriers allow bacteria to enter the bloodstream during routine activities like chewing and brushing, leading to transient bacteremia [19]. In periodontitis, inflammation compromises the gingival epithelial barrier by disrupting tight junctions, a process mediated by factors like LPS [20]. This permits oral bacteria and their LPS to translocate into the bloodstream via the paracellular route during routine activities such as mastication, resulting in transient bacteremia. Such process highlights how localized periodontal infection can become a systemic concern.

Given the complexity and multidimensional nature of sepsis pathogenesis, the current therapeutic paradigm is undergoing a profound shift—from a narrow focus on antimicrobial activity toward an integrated strategy that emphasizes immunomodulation and detoxification [21,22]. An ideal modern sepsis therapeutic should possess multiple synergistic functions: it should not only eliminate pathogens effectively but also neutralize their toxins, clear endogenous danger signals, and modulate the excessive host inflammatory response [23]. However, achieving this goal poses significant challenges[24]. First, simply combining different functional units—such as antimicrobials, nucleic acid scavengers, and antioxidants—may fail to achieve synergistic effects at the disease site due to divergent pharmacokinetics [25]. Second, conventional drug molecules are often inefficient at capturing and neutralizing high levels of cfDNA in the bloodstream [26]. Furthermore, issues related to targeting specificity and biosafety under systemic administration cannot be overlooked. Therefore, developing a single platform capable of integrating multiple therapeutic functions and enabling spatiotemporal synergy is critical to overcoming current treatment bottlenecks. This is precisely where nanomedicine can leverage its unique advantages in functional integration.

Given the multifaceted pathogenesis of sepsis, a single therapeutic modality cannot adequately address the concurrent challenges of bacterial endotoxins, cfDNA accumulation, and oxidative injury; this necessitates the development of an integrated nanoplatform capable of synergistic detoxification and immunomodulation. Herein, we constructed a multifunctional nanosystem (HPB-NPs@PME@PEI) based on hollow Prussian blue nanoparticles loaded with polymyxin E and surface-modified with polyethylenimine, and we hypothesize that this platform can concurrently neutralize LPS, scavenge cfDNA, and eliminate ROS, thereby blocking the TLR4/MyD88/NF-κB inflammatory cascade and significantly improving survival outcomes in murine sepsis models.

## 2 Results

### 2.1 Preparation and characterization of PME@HPB@PEI nanozymes

**Fig. 1:**
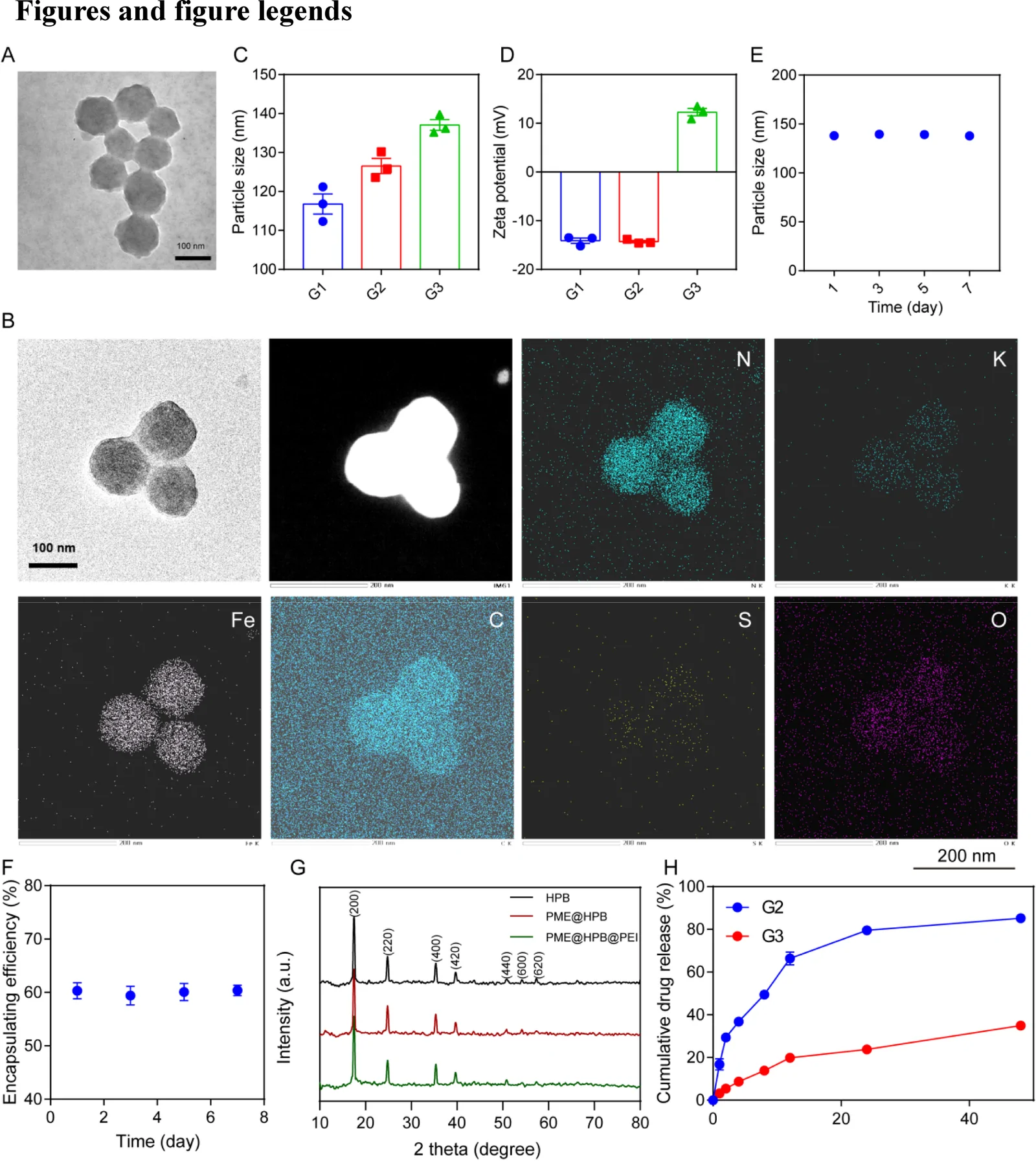
Preparation and characterization of PME@HPB@PEI nanozymes. (a) TEM image of PME@HPB@PEI nanozymes. (b) TEM assisted element mapping of PME@HPB@PEI nanozymes. (c) Particle sizes of different nanoparticles. (d) Zeta potentials of different nanoparticles. (e) Particle size stability of PME@HPB@PEI nanozymes. (f) Encapsulating efficiency stability of PME@HPB@PEI nanozymes. (g) XRD of different nanoparticles. (h) PME releases of PME@HPB and PME@HPB@PEI nanozymes. G1: PME; G2: PME@HPB; G3: PME@HPB@PEI nanozymes.

To achieve concurrent antibacterial activity, LPS neutralization, cfDNA scavenging, ROS elimination, and anti-inflammatory effects, we synthesized PME@HPB@PEI nanozymes. TEM results revealed that both HPB and PME@HPB nanoparticles exhibit well-dispersed nanostructures (Fig. 1A and Fig. S1). The PME@HPB@PEI nanozymes also maintained a well-dispersed morphology, with a polymer layer observable on the nanoparticle surface, confirming successful PEI modification (Fig. 1A). Furthermore, TEM-based elemental mapping demonstrated successful nanoparticle synthesis: the presence of iron and nitrogen elements, characteristic of HPB, and sulfur originating from sulfate groups in PME (Fig. 1B). Dynamic light scattering measurements indicated a slight increase in the hydrodynamic size of PME@HPB@PEI nanozymes compared to PME and PME@HPB nanoparticles (Fig. 1C). Similarly, while both PME and PME@HPB nanoparticles exhibited negative surface charges, the PME@HPB@PEI nanozymes showed a positive zeta potential, verifying successful modification with positively charged PEI (Fig. 1D).

We next evaluated the stability of the nanoparticles. Over one week of storage, the particle size, surface potential, and PDI of PME@HPB@PEI nanozymes showed minimal fluctuations, demonstrating good stability suitable for biomedical applications (Fig. 1E and Fig. S2). Additionally, we determined the encapsulation efficiency and drug loading efficiency of PME in the PME@HPB@PEI nanozymes. The encapsulation efficiency exceeded 60%, and the drug loading efficiency was greater than 20% (Fig. 1F). Moreover, both parameters remained largely unchanged during one week of storage, indicating no significant drug leakage (Fig. 1F and Fig. S3).

To further analyze the synthesis of the nanozymes, XRD patterns were collected. The results showed that the characteristic diffraction peaks of PME@HPB@PEI nanozymes were similar to those of PME@HPB and pure HPB, confirming that neither antibiotic loading nor PEI modification disrupted the crystal structure of HPB (Fig. 1G). Finally, the antibiotic release profile of PME@HPB@PEI nanozymes was evaluated. Within 48 h, approximately 80% of the loaded PME was released from PME@HPB nanoparticles, whereas the release from PME@HPB@PEI nanozymes was only about 30% (Fig. 1H). This demonstrates that PEI modification enables sustained antibiotic release, which is expected to enhance both antibacterial efficacy and LPS neutralization capacity.

### 2.2 *In vitro* characterization of PME@HPB@PEI nanozymes

**Fig. 2:**
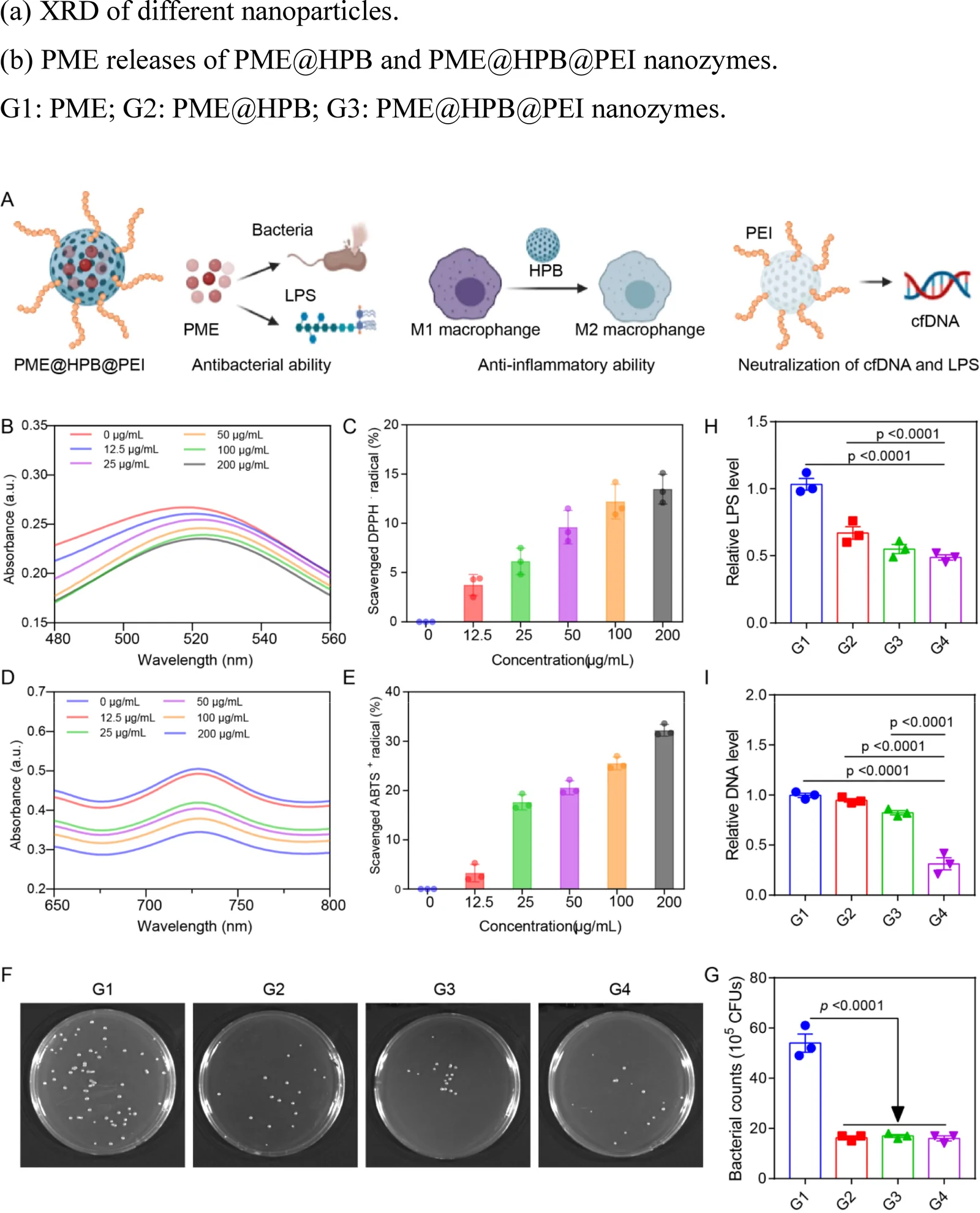
*In vitro* characterization of PME@HPB@PEI nanozymes. (a) Schematic illustration of PME@HPB@PEI nanozymes: antibacterial ability, anti-inflammatory ability, and neutralization of LPS and cfDNA. (b, c) DPPH scavenging efficiency of different concentrations of PME@HPB@PEI nanozymes. (d, e) ABTS scavenging efficiency of different concentrations of PME@HPB@PEI nanozymes. (f) Bacterial colonies on LB plates in different groups. (g) Quantification of bacterial colonies in different groups. (h) Neutralization of LPS: relative LPS level in different groups. (i) Neutralization of cfDNA: relative cfDNA level in different groups.

The PME@HPB@PEI nanozyme exhibits integrated capabilities for ROS scavenging, anti-inflammatory activity, antibacterial action, and neutralization of LPS and cell-free DNA (cfDNA) (Fig. 2A). First, we evaluated the ROS-scavenging capacity of the PME@HPB@PEI nanozyme. Both DPPH and ABTS assays demonstrated its significant ability to eliminate reactive oxygen species (Fig. 2B–E). Moreover, this ROS-scavenging effect was concentration-dependent, with higher nanozyme concentrations leading to greater clearance efficiency (Fig. 2B–E). We next assessed the antibacterial performance of the PME@HPB@PEI nanozyme using *Escherichia coli*—a common pathogen in sepsis—as a model strain. The results confirmed its potent antimicrobial activity (Fig. 2F and G). Given that PME is known to neutralize LPS, we further examined the endotoxin-neutralizing capacity of the nanozyme. The data indicated that PME@HPB@PEI effectively neutralized LPS. Finally, we evaluated the ability of the nanozyme to scavenge cell-free DNA (Fig. 2H). While neither PME alone nor PME@HPB showed notable cfDNA-neutralizing activity, the PME@HPB@PEI nanozyme significantly reduced cfDNA levels in the system (Fig. 2I). This can be attributed to the positively charged PEI on the nanozyme surface, which electrostatically adsorbs and neutralizes negatively charged cfDNA.

To evaluate the *in vivo* biodistribution and targeting efficiency of the nanoplatform, we tracked Cy5-labeled HPB-NPs@PME@PEI in CLP-induced septic mice. As shown in Fig. S4, the fluorescence signal of Cy5-HPB-NPs@PME@PEI was detected in major organs including the intestine, kidney, liver, and lung at 12 (Fig. S4A), 24 (Fig. S4B), and 48 h (Fig. S4C) post-injection, with peak accumulation observed at 24 h in most organs. Notably, the lung and kidney—the primary target organs in sepsis—exhibited the highest fluorescence intensity among all tested tissues, indicating preferential distribution to these injury sites. We next evaluated the long-term biosafety of HPB-NPs@PME@PEI in healthy mice, with representative data at day 7 presented in Fig. S5. Mice receiving a single intravenous injection of the nanoplatform (5 mg/kg PME equivalent) showed no significant differences in serum biochemical parameters, including ALT, AST, BUN, and CRE (Fig. S5A–D), compared to the PBS-treated sham group, indicating no detectable hepatotoxicity or nephrotoxicity. Furthermore, serum levels of pro-inflammatory cytokines (TNF-α, IL-6, IL-1β) (Fig. S5E–G) and complement activation markers (C3a, C5a) (Fig. S5H–I) remained comparable between the two groups, demonstrating that the PEI-modified nanoplatform does not elicit significant immunogenicity or complement-mediated inflammatory responses. Collectively, these findings demonstrate that the PME@HPB@PEI nanozyme effectively addresses multiple pathological factors in sepsis—including bacterial infection, elevated LPS and cfDNA levels, and ROS accumulation—through a multifunctional mechanism.

### 2.3 HPB-NPs@PME@PEI ameliorate systemic injury in CLP-induced sepsis

**Fig. 3:**
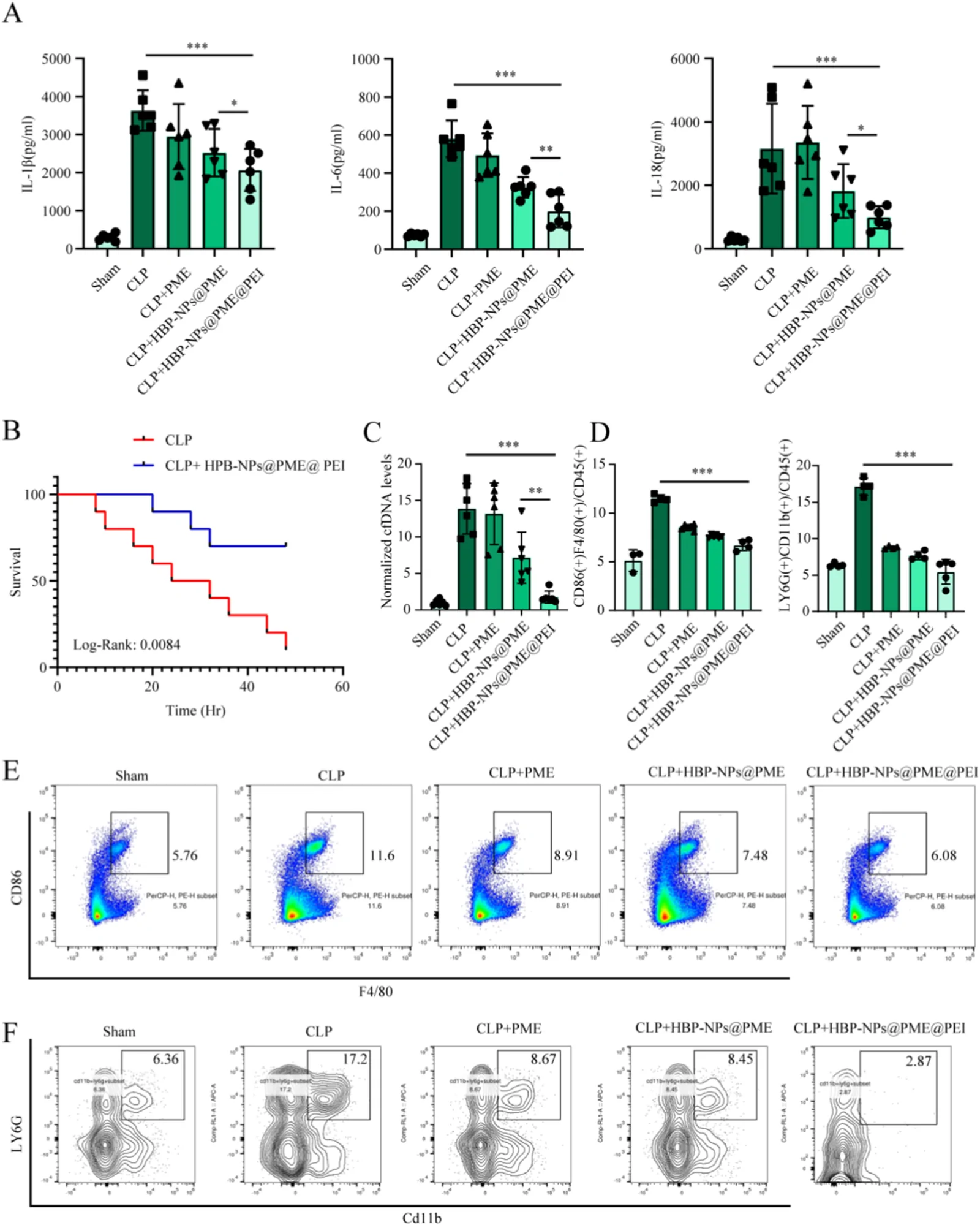
HPB-NPs@PME@PEI ameliorate systemic injury in CLP-induced sepsis. (a) Plasma levels of pro-inflammatory cytokines at 24 h post-CLP. (b) Survival curves of mice in different treatment groups following CLP. (c) Relative levels of circulating cfDNA in plasma. (d-f) Flow cytometry analysis showing CLP-induced increases in CD86(+)F4/80(+) macrophages and CD11b(+)LY6G(+) neutrophils and corresponding quantification.

Survival analysis and assessment of systemic inflammatory and cfDNA burden were performed in a murine cecal ligation and puncture (CLP) model. Mice were treated with PBS, PME, HPB-NPs@PME, or the final formulation HPB-NPs@PME@PEI; sham-operated animals served as controls. The CLP-induced plasma levels of pro-inflammatory cytokine elevation was most effectively suppressed by HPB-NPs@PME@PEI (Fig. 3A). The survival rate was significantly improved in the HPB-NPs@PME@PEI group compared to the CLP and PME-only groups (Fig. 3B), underscoring the therapeutic efficacy of the integrated nanosystem. Concordantly, circulating cfDNA was markedly elevated in the CLP group (Fig. 3C). While free PME showed a modest reduction, treatment with HPB-NPs@PME@PEI most effectively suppressed these parameters, restoring them to sham levels. To further study the immune activation, we employed flow cytometry for macrophage and neutrophil analysis. CLP triggered significant elevation in two innate immune cell types (Fig. 3D–F), which were reversed to different extent to normal levels in HPB-NPs@PME@PEI treatment group. These results indicate that our nanosystem potently mitigates the systemic inflammatory storm and reduces the burden of pathogenic cfDNA in sepsis.

### 2.4 Therapeutic efficacy in LPS-induced endotoxic shock model

To validate the therapeutic efficacy of HPB-NPs@PME@PEI in a complementary sepsis model with distinct pathophysiology, we established an LPS-induced endotoxic shock model (Fig. S6). Mice receiving LPS challenge (10 mg/kg) exhibited markedly elevated serum levels of pro-inflammatory cytokines including TNF-α, IL-6, IL-1β, and IFN-γ, along with a compensatory increase in the anti-inflammatory cytokine IL-10, as measured by ELISA. Treatment with HPB-NPs@PME@PEI significantly attenuated the production of all tested pro-inflammatory cytokines while restoring IL-10 to near-normal levels (Fig. S6A–E). Consistent with the reduction in systemic inflammation, the nanoplatform also effectively reduced circulating cfDNA levels and MPO-dsDNA complexes, indicating suppression of NETosis and reduced cellular damage (Fig. S6F–G). Serum biochemical analysis revealed that LPS challenge induced significant elevation of hepatic (ALT, AST), renal (BUN, CRE), and general cellular injury (LDH) markers, all of which were markedly reduced by HPB-NPs@PME@PEI treatment (Fig. S6H–L). Immunofluorescence staining further demonstrated that the nanoplatform substantially reduced infiltration of LY6G^+^ neutrophils and F4/80^+^ macrophages in the lung, kidney, and liver of LPS-challenged mice (Fig. S6M–O). Moreover, Western blot analysis of liver tissues showed that HPB-NPs@PME@PEI downregulated the expression of iNOS, CD86, and PAD4—key markers of inflammatory macrophage polarization and neutrophil activation—compared to the LPS alone group (Fig. S6P–Q). Taken together, these results confirm that the nanoplatform exerts broad-spectrum protective effects against systemic inflammation and multi-organ injury in the LPS-induced endotoxic shock model.

### 2.5 HPB-NPs@PME@PEI attenuate CLP-induced kidney injury

**Fig. 4:**
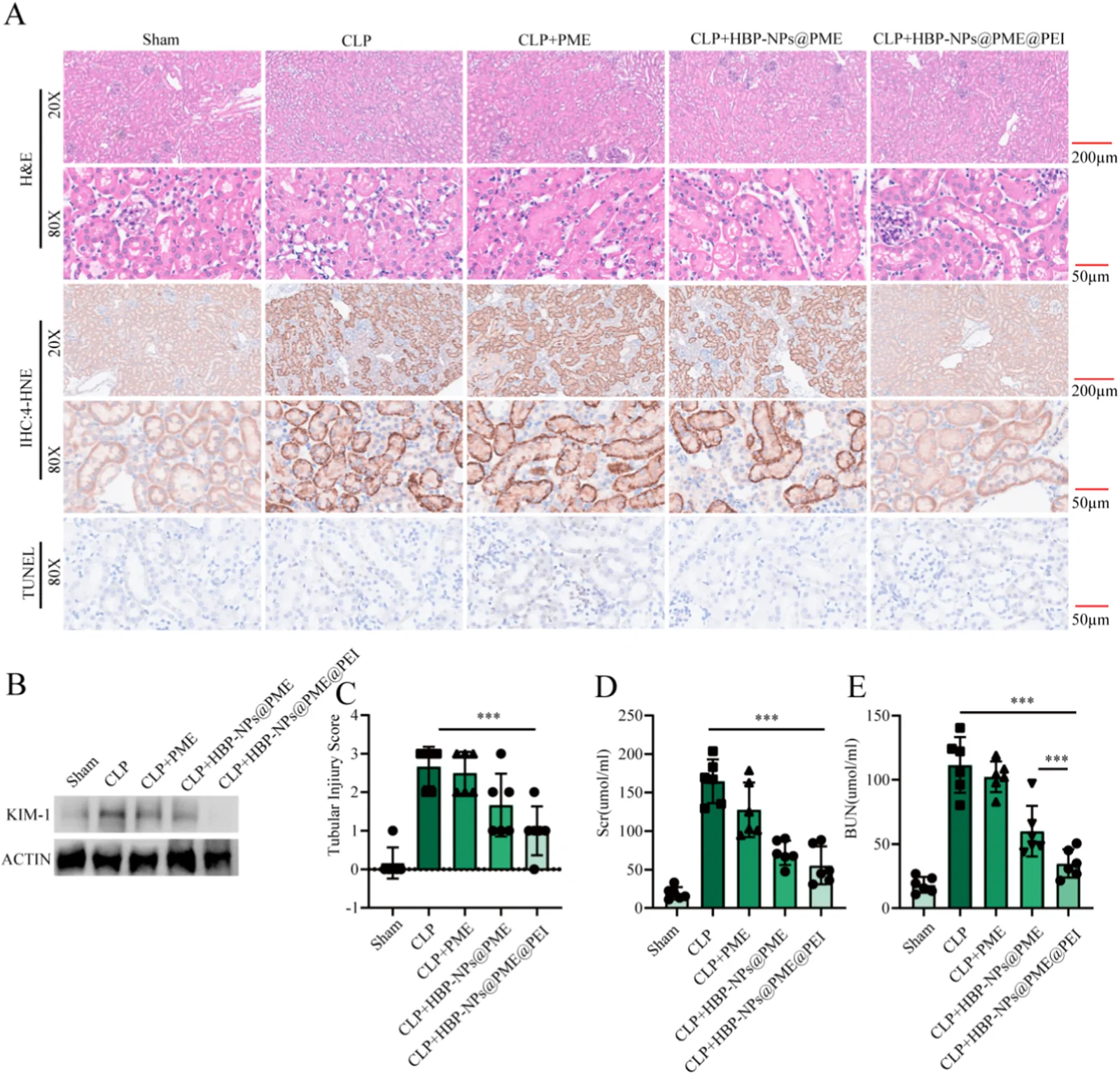
HPB-NPs@PME@PEI attenuate CLP-induced kidney injury. (a) Representative H&E, IHC (4-HNE), and TUNEL staining of kidney tissues. (b, c) Relative protein expression level of renal injury marker KIM-1, assessed by WB analysis (b) and quantification of tubular injury score (c). (d) Serum levels of kidney function markers Serum creatinine (Scr) and blood urea nitrogen (BUN).

Renal function and pathology were evaluated post-CLP. The CLP group exhibited severe renal impairment, as evidenced by significantly elevated serum creatinine and blood urea nitrogen (BUN), alongside upregulated expression of the renal injury marker Kidney Injury Molecule-1 (KIM-1). H&E and IHC analysis suggested that treatment with HPB-NPs@PME@PEI dramatically reduced 4-HNE accumulation in injured renal tubular cells and lowered the nuclear TUNEL positive rate (Fig. 4A). Among different HPB-NPs-based regiments, HPB-NPs@PME@PEI most efficiently suppressed CLP-induced KIM-1 expression elevation and the tubular injury (Fig. 4B and C). Regarding the impaired renal function, we also observed most efficient rescue of down-regulated renal function indicators, including Scr and BUN levels (Fig. 4D). These improvements were significantly superior to those in the free PME or HPB-NPs@PME groups, demonstrating the superior reno-protective effects of the PEI-modified nano-system through combined anti-inflammatory activities.

### 2.6 HPB-NPs@PME@PEI alleviate CLP-induced lung injury

**Fig. 5:**
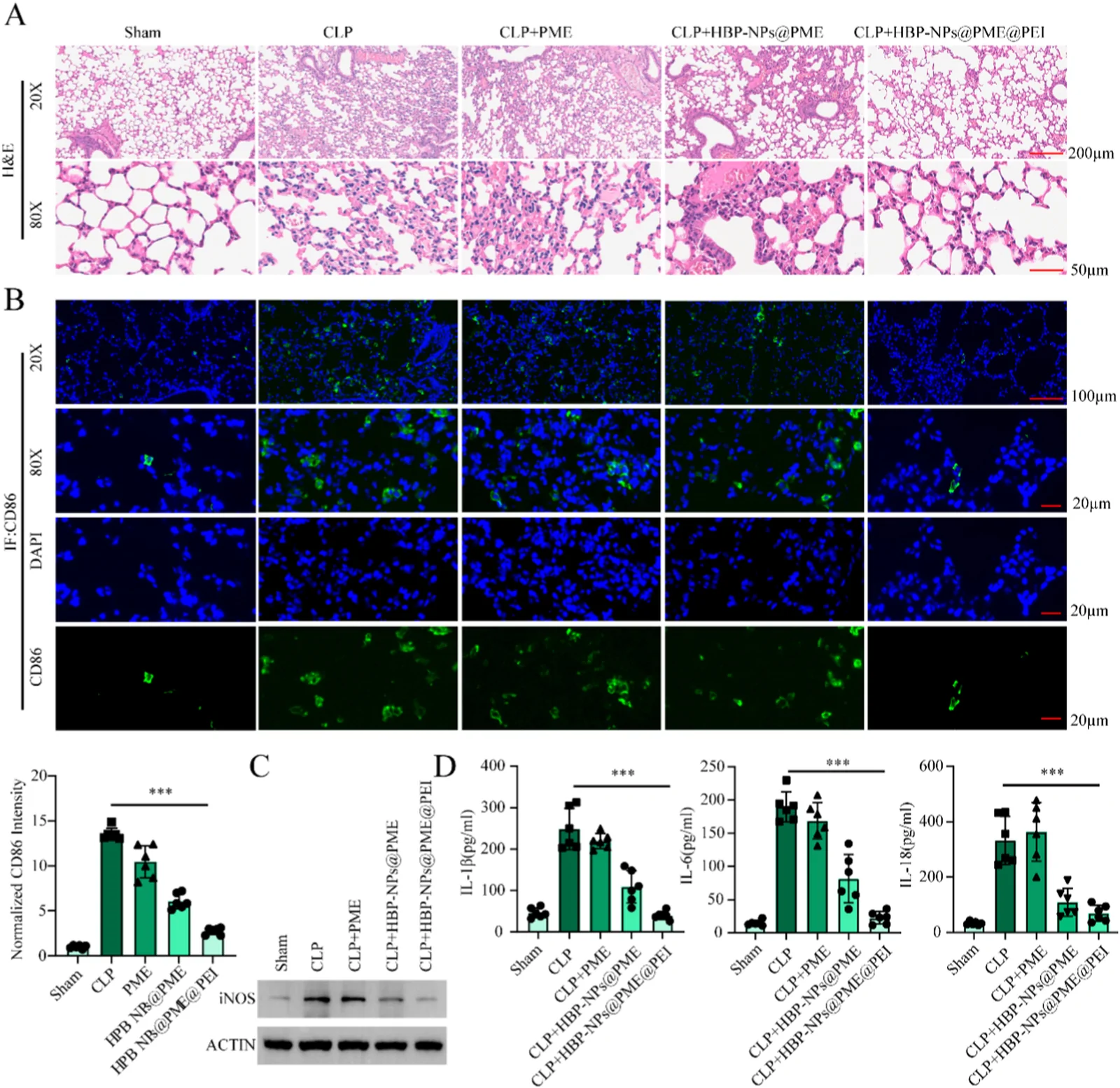
HPB-NPs@PME@PEI alleviate CLP-induced lung injury. (a) Representative H&E staining of lung tissues. (b) Immunofluorescence staining of macrophage (CD86^+^) infiltration and quantification of CD86 positive intensity in lung sections. (c) Western blot analysis of iNOS protein expression in lung tissue. (d) Levels of pro-inflammatory cytokines in bronchoalveolar lavage fluid.

Various histopathological examination approaches of lung tissues showed severe alveolar damage, inflammatory cell infiltration, and interstitial thickening in the CLP group. H&E analysis suggested that the pathological changes were considerably ameliorated by HPB-NPs@PME@PEI treatment (Fig. 5A). Consistent with the histological findings, immunofluorescence analysis confirmed a significant reduction in inflammatory macrophage infiltration in the lungs of the nanosystem-treated group (Fig. 5B). Western blot analysis revealed that the upregulation of iNOS in lung tissue post-CLP was effectively suppressed by HPB-NPs@PME@PEI (Fig. 5C). Furthermore, bronchoalveolar lavage fluid (BALF) from these mice showed markedly lower levels of IL-1β, IL-6, and IL-18 (Fig. 5D). These data collectively indicate that our nanosystem mitigates lung injury by curbing inflammatory cell recruitment and cytokine release.

### 2.7 HPB-NPs@PME@PEI inhibit LPS-induced activation of innate immune cells *in vitro*

**Fig. 6:**
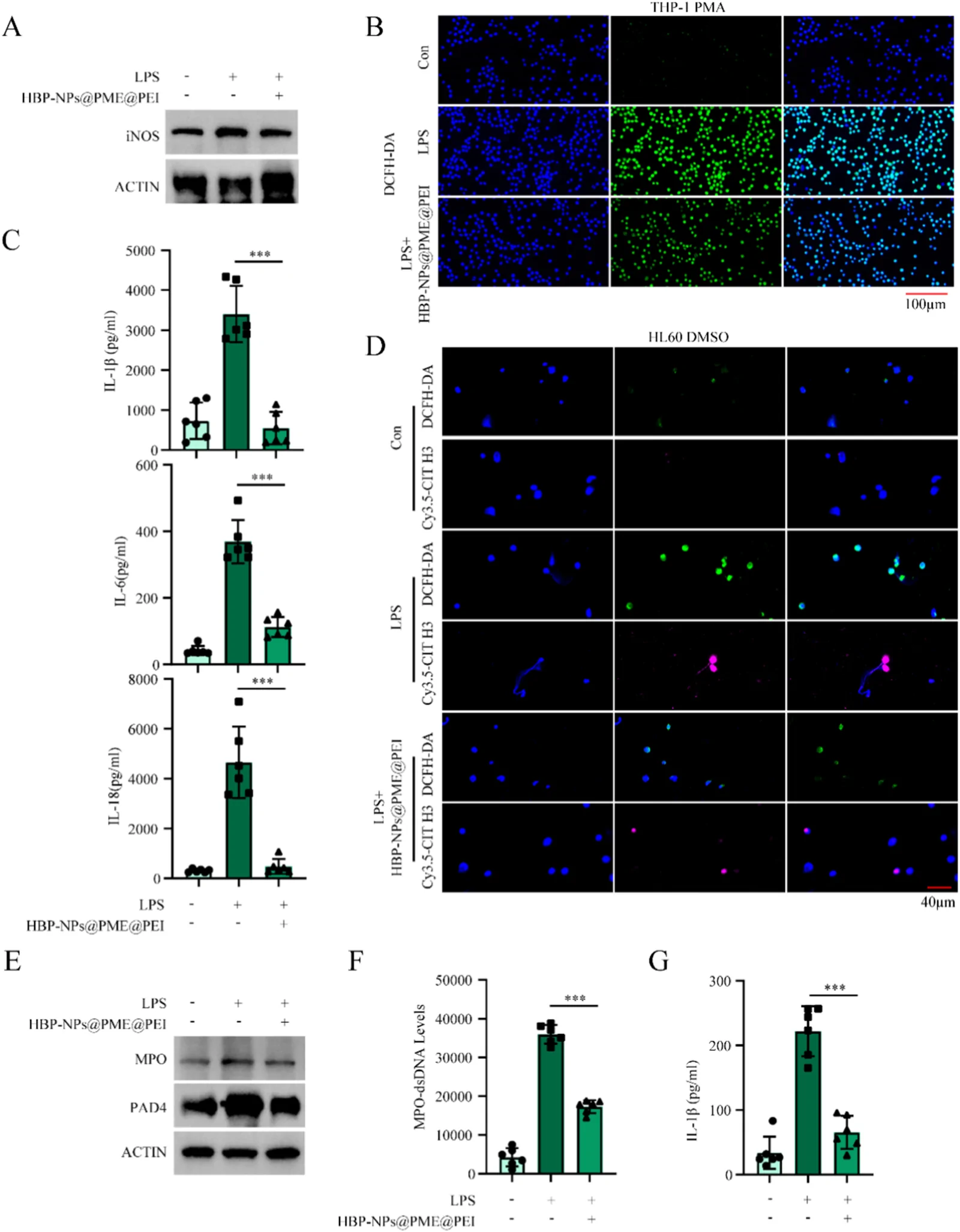
HPB-NPs@PME@PEI inhibit LPS-induced activation of innate immune cells *in vitro*. (a) Western blot analysis of iNOS expression in PMA-induced THP-1 macrophages. (b) Intracellular ROS levels measured by DCFH-DA fluorescence in PMA-induced THP-1 cells. (c) Secretion levels of IL-1β, IL-6, and IL-18 from THP-1 cells. (d) DCFH-DA fluorescence and immunofluorescence of citrullinated histone H3 (Cit-H3) in DMSO-induced HL-60 cells. (e) Western blot analysis of PAD4 and MPO expression in HL-60 cells. (f) Relative levels of MPO-dsDNA complexes in cell culture supernatant. (g) Secretion of IL-1β from HL-60 cells.

To study the role of HPB-NPs@PME@PEI in reducing macrophage-originated inflammation, we opted for the human macrophage cell line with/without LPS stimulation for further analysis. LPS stimulation significantly upregulated iNOS protein expression in the THP-1 cells induced with PMA and LPS (Fig. 6A). We observed the HPB-NPs@PME@PEI nanoparticles effectively reversed up-regulation of DCFH-DA accumulation in the LPS-treated group (Fig. 6B). LPS treatment increased the secretion of IL-1β, IL-6, and IL-18 in the THP-1 cells, which was efficiently reversed by HPB-NPs@PME@PEI nanoparticles (Fig. 6C). To dissect the mechanism underlying the anti-inflammatory effect, we used an LPS-stimulated human promyelocytic leukemia (HL-60) cell model with DMSO induction. Co-incubation with HPB-NPs@PME@PEI nanoparticles effectively reversed all these changes, reducing DCFH-DA accumulation and cit-H3 fluorescence intensity (Fig. 6D). Western blot analysis further demonstrated that the nanoparticles suppressed the LPS-induced expression of PAD4 and MPO activation (Fig. 6E). LPS enhanced the formation of MPO-dsDNA complexes and IL-1β secretion, which were reduced by HPB-NPs@PME@PEI treatments (Fig. 6F and G). These *in vitro* results confirm that the HPB-NPs@PME@PEI nano-system directly inhibits LPS-triggered inflammatory activation and NETosis in innate immune cells.

### 2.8 HPB-NPs@PME@PEI protect lung epithelial cells from LPS-induced injury by inhibiting the TLR4/NF-κB pathway

**Fig. 7:**
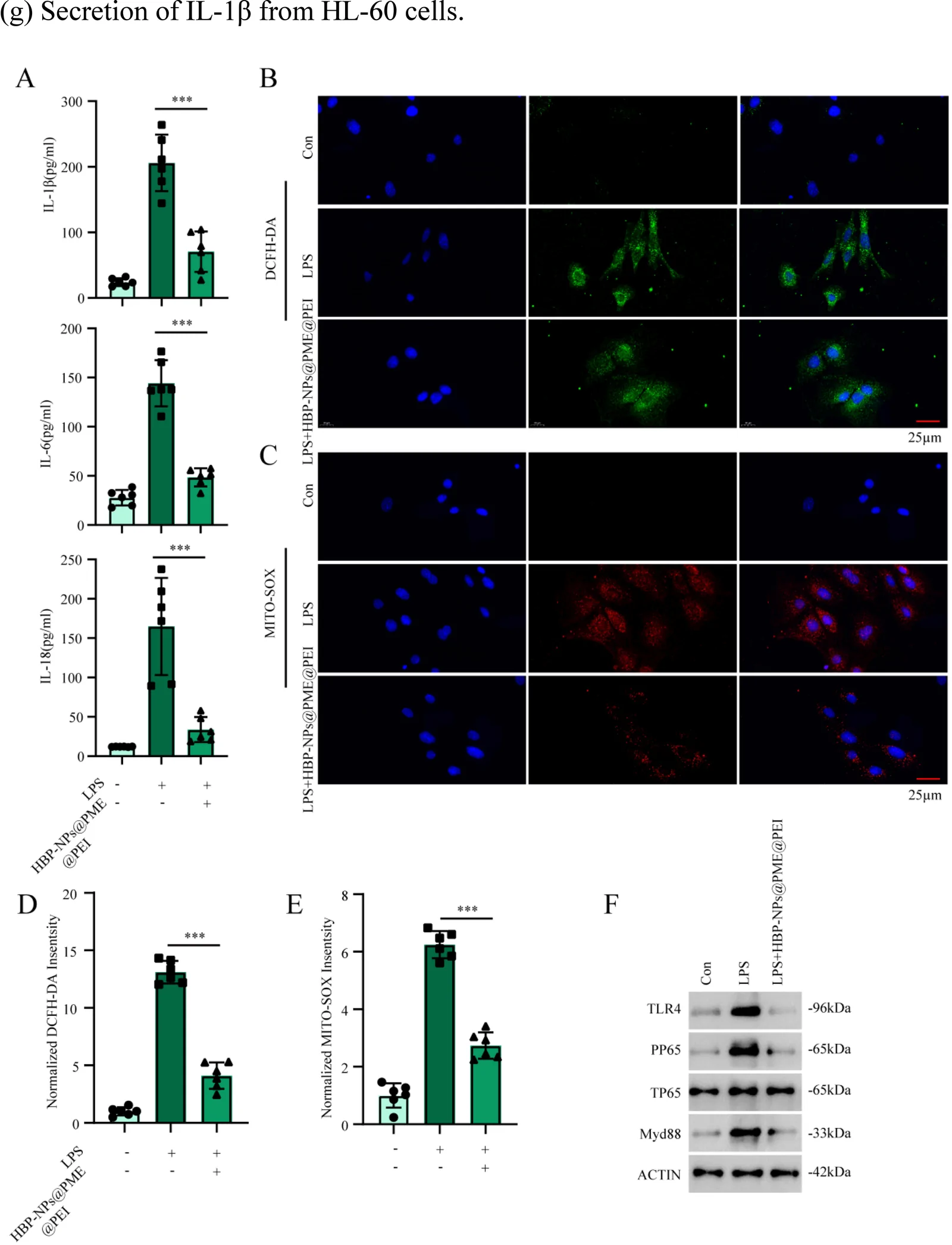
HPB-NPs@PME@PEI protect lung epithelial cells from LPS-induced injury by inhibiting the TLR4/NF-κB pathway. (a) Secretion levels of IL-1β, IL-6, and IL-18 from lung epithelial cells. (b, c) Measurement of total intracellular ROS and mitochondrial ROS (mito-SOX) levels. (d, e) Representative fluorescence quantification of DCFH-DA and Mito-SOX signals. (f) Western blot analysis of key proteins in the TLR4/MyD88/NF-κB signaling pathway.

In LPS-stimulated lung epithelial cells, HPB-NPs@PME@PEI treatment significantly reduced the secretion of IL-1β, IL-6, and IL-18 (Fig. 7A). We observed HPB-NPs@PME@PEI lowered LPS-induced ROS accumulation (Fig. 7B) and intra-mitochondrial ROS accumulation (Fig. 7C), which were validated by DCFH-DA and mito-SOX intensity signals (Fig. 7D and E). Mechanistic investigation revealed that the HPB-NPs@PME@PEI nanoparticles inhibited the activation of the TLR4/MyD88/NF-κB signaling axis (Fig. 7F). These findings suggest that the protective effect against lung epithelial cell injury is mediated through simultaneous LPS neutralization, ROS clearance, and inhibition of the TLR4/NF-κB inflammatory pathway.

### 2.9 HPB-NPs@PME@PEI ameliorate LPS-induced renal tubular epithelial cell injury via suppression of the TLR4/NF-κB pathway

**Fig. 8:**
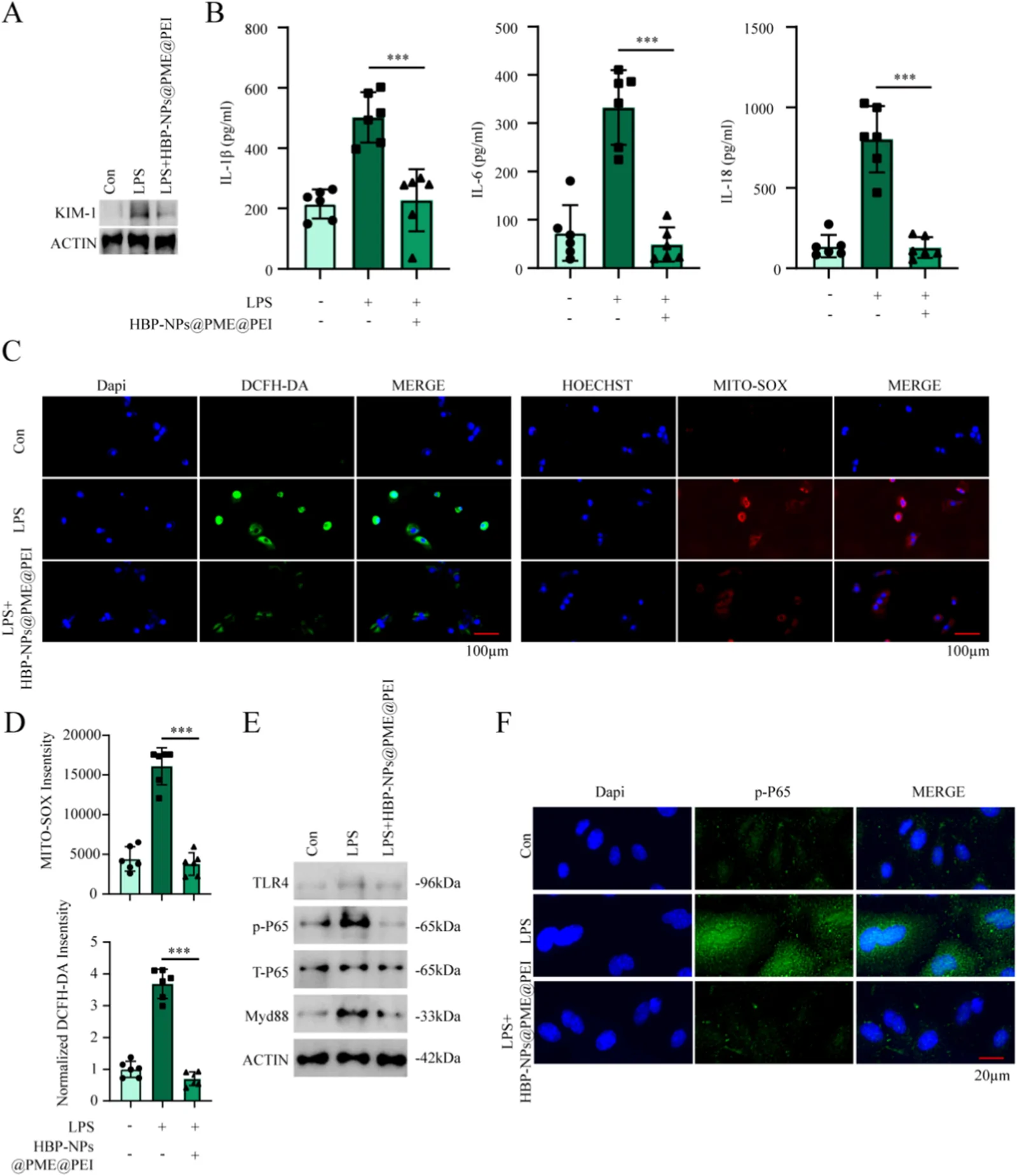
HPB-NPs@PME@PEI ameliorate LPS-induced renal tubular epithelial cell injury via suppression of the TLR4/NF-κB pathway. (a) Expression level of renal injury marker KIM-1. (b) Secretion levels of IL-1β, IL-6, and IL-18 in the cultured HK-2 renal tubular cells. (c, d) Representative fluorescence images and quantification of DCFH-DA and Mito-SOX signals. (e) Western blot analysis of TLR4, MyD88, and p-p65 protein expression. (f) Immunofluorescence staining and quantification of p65 nuclear translocation.

Similar protective effects and mechanisms were observed in renal tubular epithelial cells. LPS stimulation led to elevated KIM-1 expression (Fig. 8A), increased inflammatory cytokine release (Fig. 8B). Treatment with HPB-NPs@PME@PEI nanoparticles effectively mitigated elevation of DCFH-DA and mito-SOX signal (Fig. 8C and D). WB analysis indicated higher TLR4/MyD88/NF-κB activation (Fig. 8E), accompanied by enhanced p65 nuclear translocation (Fig. 8F). The results demonstrate that the HPB-NPs@PME@PEI confers protection to renal tubular cells by neutralizing LPS, scavenging ROS, and disrupting the key TLR4/NF-κB signaling cascade, thereby reducing inflammatory injury.

### 2.10 HPB-NPs@PME@PEI suppresses cfDNA-induced STING pathway activation and inflammatory responses in human primary cells

To further elucidate the molecular mechanism by which PEI-mediated cfDNA scavenging suppresses inflammation, we investigated the cGAS-STING pathway—a primary cytosolic DNA sensing axis—in HK-2 renal epithelial cells and THP-1-derived macrophages. As shown in Fig. S7A–B, cfDNA stimulation significantly upregulated the expression of cGAS and STING in both cell types, indicating robust activation of the STING pathway. Co-incubation with PEI effectively reversed these changes, reducing STING levels as well as downstream IFN-β production. Treatment with HPB-NPs@PME yielded little inhibitory effects, confirming that the positively charged PEI is the key functional component responsible for cfDNA scavenging and subsequent STING pathway blockade. Consistent with the Western blot findings, ELISA quantification of IFN-β in culture supernatants further confirmed that PEI alone, but not HPB-NPs@PME, significantly reduced IFN-β secretion in both HK-2 and THP-1 cells (Fig. S7C–D). To validate the translational relevance of our findings, we extended our investigation to human primary cells. Primary macrophages derived from human peripheral blood mononuclear cells were stimulated with LPS in the presence or absence of HPB-NPs@PME@PEI. As shown in Fig. S7E, the nanoplatform significantly reduced the secretion of TNF-α, IL-6, and IL-1β, and concurrently decreased intracellular ROS accumulation as reflected by DCFH-DA fluorescence intensity. Similarly, in primary human neutrophils isolated from peripheral blood, HPB-NPs@PME@PEI treatment markedly suppressed LPS-induced production of TNF-α, IL-6, and IL-1β, along with reduced ROS levels (Fig. S7F). Collectively, these results demonstrate that HPB-NPs@PME@PEI inhibits the cfDNA-induced cGAS-STING pathway—in addition to the previously characterized TLR4/NF-κB axis—and effectively suppresses inflammatory responses in human primary macrophages and neutrophils, strongly supporting its clinical translational potential.

## 3 Discussion

The multifunctional nanozyme HPB-NPs@PME@PEI was comprehensively characterized, demonstrating integrated *in vitro* capabilities for potent ROS scavenging, antibacterial action against *E. coli*, and effective neutralization of LPS and cfDNA. In a murine CLP sepsis model, HPB-NPs@PME@PEI significantly improved survival, outperforming free PME, by drastically reducing systemic inflammation, cfDNA, and NETs markers, while also alleviating kidney and lung injury as shown by restored histology and function. Mechanistic *in vitro* studies revealed that the platform directly inhibits LPS-induced inflammatory activation and NETosis in macrophages and neutrophils, and protects lung and renal epithelial cells by concurrently scavenging ROS and suppressing the critical TLR4/MyD88/NF-κB signaling pathway, confirming its synergistic multi-target therapeutic action. We acknowledge that experimental plasma/serum stability studies were not performed. Nevertheless, several physicochemical properties of HPB-NPs@PME@PEI support its expected colloidal stability in biological fluids: a hydrodynamic size of ∼125 nm with narrow distribution (PDI < 0.2), a positive zeta potential of +32 mV (Fig. 1C and D) providing electrostatic repulsion, and PEI-mediated steric stabilization that minimizes opsonization. These predictions are indirectly supported by the sustained PME release profile (∼30% over 48 h) and minimal fluctuations in particle size and PDI during one-week PBS storage. However, we acknowledge that direct experimental validation of plasma stability—including particle size/zeta potential changes over time, drug leakage in biological matrices, and protein corona composition—remains essential and will be addressed in future studies.

Macrophages, as central regulators of innate and adaptive immunity, exhibit critical heterogeneity and plasticity in their response during sepsis. The progression of sepsis is heavily influenced by an imbalance between pro-inflammatory M1-like and anti-inflammatory M2-like macrophage polarization, which disrupts the host's immune equilibrium [27]. Metabolic reprogramming in macrophages—characterized by increased glycolysis and succinate accumulation—drives cardiac inflammation and injury via HIF-1α stabilization and NLRP3 inflammasome activation. Natural compound, chicoric acid (CA), protects against septic cardiomyopathy by specifically inhibiting the key metabolic enzyme succinate dehydrogenase (SDH) in macrophages, thereby reducing glycolysis, succinate levels, and subsequent NLRP3 activation [28]. In early sepsis, increased lysophosphatidylcholine (LPC) induced expression of the costimulatory molecule GITR on macrophages, which in turn exacerbates systemic inflammation by specifically enhancing NLRP3 inflammasome-mediated pyroptosis. Mechanistically, macrophage GITR promotes NLRP3 activation by competing with it for the E3 ligase MARCH7 and by recruiting MARCH7 to degrade the deacetylase SIRT2, thereby increasing NLRP3 acetylation and reducing its ubiquitination [29]. In sepsis, neutrophils serve as critical first responders, deploying antimicrobial defenses including the release of NETs. However, excessive NET release contributes to endothelial dysfunction by promoting a pro-inflammatory and pro-coagulant state, degrading the protective glycocalyx, and increasing vascular permeability. This cascade of neutrophil-mediated damage ultimately leads to microcirculatory failure and life-threatening organ dysfunction in the late stages of sepsis [30]. Upregulated PD-L1 on neutrophils acts as a critical survival signal, directly delaying apoptosis to exacerbate inflammation. This effect is mediated through PD-L1's interaction with PI3K, which activates the AKT-dependent anti-apoptotic pathway. Consequently, genetic deletion of neutrophil PD-L1 reduces lung injury, improves cytokine balance, and lowers mortality in experimental sepsis, highlighting it as a pathogenic driver [31]. Thus, macrophages and neutrophils drive multi-organ failure through a synergistic pathogenic alliance, where macrophage polarization imbalance and metabolically-activated NLRP3 inflammasomes converge to create a self-amplifying cycle of inflammation and endothelial dysfunction.

The dysregulated activation of the TLR4/MyD88/NF-κB signaling pathway serves as a central convergence point in sepsis pathology, being triggered by distinct upstream regulators that amplify inflammation and drive organ injury. In sepsis, researchers found that increased PCSK9 expression during sepsis activates this inflammatory pathway, exacerbating vascular injury and reducing survival in mice. Increased PCSK9 expression during sepsis directly activates the TLR4/MyD88/NF-κB signaling pathway, which in turn triggers the NLRP3 inflammasome. This dual-pathway activation drives a potent inflammatory response, leading to vascular endothelial dysfunction [32]. Elevated expression of the protein CMTM3 on neutrophils exacerbates organ damage by dysregulating their migration. This occurs because CMTM3 upregulates TLR4 expression, which in turn promotes surface expression of the chemokine receptor CXCR2 via the MyD88/NF-κB signaling pathway, driving excessive neutrophil mobilization from bone marrow to organs [33]. The sphingolipid sphinganine is a critical lipid checkpoint for initiating TLR4/MyD88/NF-κB signaling by physically interacting with and promoting the membrane recruitment of the adaptor protein MyD88 in macrophages. Disrupting sphinganine biosynthesis in a sepsis model inhibits this MyD88 recruitment, thereby dampening the downstream inflammatory response [34]. Collectively, recent research elucidates that sepsis progression is driven by the activation of the central TLR4/MyD88/NF-κB inflammatory pathway through distinct upstream mechanisms, highlighting the pathway's multifaceted role in orchestrating multi-organ injury. In our study, the developed HPB-NPs@PME@PEI nanosystem provides direct protection to both lung and renal epithelial cells against LPS-induced injury. This protection is achieved through a dual mechanism: scavenging harmful ROS and concurrently inhibiting the activation of the TLR4/MyD88/NF-κB signaling pathway. By simultaneously blocking this major inflammatory cascade and alleviating oxidative stress, the treatment effectively reduces cytokine release and mitigates cellular damage in both organ systems.

In the context of sepsis-induced acute lung injury, TLR4 signaling critically regulates macrophage immunometabolism, where TLR4 deficiency promotes an anti-inflammatory M2 phenotype by enhancing cholesterol efflux and reducing glycolysis. These altered macrophages then attenuate damage to pulmonary ECs and lymphatic ECs, with the overall inflammatory injury being driven by TLR4/MyD88/NF-κB pathway activation in both the macrophages themselves and the endothelial cells [35]. Deubiquitinating enzyme CYLD was shown to modulate the TLR4 signaling pathway to protect against sepsis-induced ALI by directly binding to the sNASP/TRAF6 axis. Specifically, CYLD prevents TRAF6 activation; when TLR4 is triggered, sNASP phosphorylation releases CYLD, allowing TRAF6 autoubiquitination and pro-inflammatory cytokine production, while dephosphorylation reforms the inhibitory complex [36]. Mechanical ventilation exacerbates sepsis-induced lung injury via a “two-hit” model, where the initial infection primes the lung for further damage. The exacerbation is driven by the caspase-1/caspase-11-HMGB1-TLR4/RAGE signaling pathway, with TLR4 playing a central role in amplifying inflammation and neutrophil recruitment [37]. Downregulation of the mitochondrial protein SIRT4 exacerbates sepsis-induced acute lung injury by amplifying the inflammatory response, mechanistically driven by the aberrant activation of the TLR4/MYD88/NF-κB signaling pathway upon SIRT4 deficiency [38]. Natural products, including terpenoids, polyphenols, alkaloids, and flavonoids, are shown to mitigate SALI by modulating key signaling pathways, with the TLR4/NF-κB pathway being a prominent target to suppress pro-inflammatory M1 activation [39]. Recent studies collectively underscore the TLR4 signaling pathway as a central regulatory hub in sepsis-induced acute lung injury, which can be therapeutically targeted through diverse strategies.

In the present study, our findings demonstrate that HPB NPs@PME@PEI directly protects renal tubular epithelial cells against LPS-induced injury, as evidenced by the downregulation of KIM-1, suppression of mitochondrial ROS, and marked inhibition of the TLR4/MyD88/NF-κB signaling cascade. This mechanistic evidence establishes the TLR4/NF-κB axis as a critical therapeutic node in sepsis-associated AKI, a notion strongly corroborated by recent studies that have identified diverse upstream drivers—including macrophage migration inhibitory factor (MIF), the immunoregulatory molecule LIGHT, and the de-ubiquitinase Usp9x—as pathogenic amplifiers of this same pro-inflammatory pathway in the septic kidney.In the context of sepsis-induced renal injury, upregulation of macrophage migration inhibitory factor (MIF) exacerbates sepsis-induced AKI by promoting NLRP3 inflammasome-mediated pyroptosis in renal cells. The mechanism is dependent on the TLR4 signaling pathway, as MIF enhances the phosphorylation of NF-κB p65, a key downstream event of TLR4 activation [40]. Immunoregulatory molecule LIGHT exacerbates inflammation and renal damage in sepsis-induced acute kidney injury, by activating the TLR4-Myd88-NF-κB signaling pathway, as evidenced by aggravated injury with recombinant LIGHT [41]. De-ubiquitinase Usp9x was found to be as a key regulator in sepsis-induced acute kidney injury by directly interacting with and stabilizing TLR4, thereby amplifying signal transduction. By removing ubiquitin from TLR4, Usp9x prevents its degradation and enhances the activation of the downstream TLR4/NF-κB pathway. This sustained signaling leads to increased inflammation and apoptosis in renal tubular cells [42]. A protective effect against sepsis-induced acute kidney injury in mice by significantly reducing inflammation and renal cell apoptosis. This renoprotection is achieved through the inhibition of key inflammatory signaling pathways, most notably the TLR4/NF-κB pathway [43]. Collectively, these studies establish the TLR4/NF-κB signaling pathway as a critical pathological nexus in sepsis-induced renal injury, which is exacerbated by various upstream regulators, but can be therapeutically targeted and protected against by agents.

Several limitations of the present study should be acknowledged. First, a direct head-to-head comparison with current first-line clinical management strategies for sepsis, such as broad-spectrum antibiotic therapy combined with fluid resuscitation or glucocorticoid treatment, was not performed. Such comparative studies would provide a more rigorous evaluation of the therapeutic advantages and translational potential of our nanoplatform and therefore represent an important direction for future investigation.

Second, the antibacterial efficacy of HPB-NPs@PME@PEI was evaluated only against *E*. *coli*. Its therapeutic activity against other clinically relevant pathogens, particularly multidrug-resistant bacteria, remains to be determined. Future studies will expand the antibacterial spectrum to include representative Gram-negative and Gram-positive pathogens, with particular emphasis on clinically prevalent carbapenem-resistant strains, and further validate the therapeutic efficacy of the nanoplatform in sepsis models induced by drug-resistant bacterial infections. Third, the physicochemical stability of HPB-NPs@PME@PEI under complex biological conditions, including plasma and serum, was not systematically investigated. Future studies will comprehensively characterize changes in particle size, zeta potential, and polydispersity index (PDI) during incubation in physiological fluids, together with drug leakage kinetics and protein corona formation. These evaluations will be essential for elucidating the *in vivo* stability, pharmacokinetic behavior, and therapeutic performance of the nanoplatform. In addition, although Western blot analyses demonstrated that treatment with HPB-NPs@PME@PEI was associated with reduced activation of the TLR4/NF-κB and STING signaling pathways, these findings are correlative rather than mechanistic. Pathway-specific validation experiments, including pharmacological inhibition or genetic silencing of TLR4, NF-κB, and STING, were not conducted in the current study. Such mechanistic investigations will be necessary to establish the causal contribution of these signaling pathways to the protective effects of the nanoplatform.

Finally, comprehensive pharmacokinetic analyses, including plasma concentration–time profiles, elimination half-life (t_1_/_2_), clearance, and area under the concentration–time curve (AUC), were not performed. The current study focused primarily on tissue biodistribution as an initial assessment of *in vivo* behavior. Nevertheless, systematic pharmacokinetic characterization will be indispensable for optimizing the dosing strategy, evaluating systemic exposure, and supporting future investigational new drug (IND)-enabling studies and clinical translation.

## 4 Conclusion

The study successfully developed and characterized a multifunctional nanozyme, HPB-NPs@PME@PEI, which integrates PME, endotoxin/DNA scavenging, and HPB properties into a single stable platform capable of neutralizing LPS, capturing cfDNA, and eliminating ROS. This engineered system demonstrated superior therapeutic efficacy in a murine sepsis model by significantly improving survival, reducing systemic inflammation and organ injury, and mechanistically protecting lung and renal cells through the concurrent scavenging of ROS and suppression of the TLR4/MyD88/NF-κB signaling pathway.

## 5 Methods

### 5.1 Synthesis of HPB nanozymes

Polyvinylpyrrolidone (PVP)-coated hollow Prussian blue (HPB) nanozymes were synthesized via a hydrothermal method. Specifically, 3 g of PVP and 131.9 mg of K_3_[Fe(CN)_6_]·3H_2_O were accurately weighed and dissolved separately in 40 mL of 0.01 M HCl solution under continuous magnetic stirring at room temperature until complete dissolution. The mixed solution was transferred to a three-neck flask and reacted in an 80 °C water bath for 20 h. After natural cooling to room temperature, the product was centrifuged to remove unreacted precursors. The precipitate was washed repeatedly with deionized water and anhydrous ethanol to obtain purified HPB nanozymes.

### 5.2 Preparation of PME@HPB nanozymes

PME-loaded HPB nanoparticles (PME@HPB) were prepared as follows: First, HPB nanoparticles were dispersed in distilled water. Subsequently, under light-protected conditions, 1 mL of PME sulfate solution (100 mM) was added dropwise to 1 mL of HPB nanoparticle suspension (2 mg). The mixture was stirred at a constant speed of 1000 rpm for 24 h, followed by centrifugation at 13,000 rpm for 15 min to collect the PME@HB nanozymes. The resulting nanoparticles were washed with distilled water to remove unbound components.

### 5.3 Fabrication of PME@HPB@PEI nanozymes

PEI polymer (Mw = 600) was mixed with PME@HB nanoparticles at a mass ratio of 1 mg (PME@HPB nanoparticles) to 200 μg (PEI) in PBS. The mixture was subjected to intermittent sonication (25% power, 3 s on/6 s off) for 10 min, followed by constant stirring at 1000 rpm for 6 h. Finally, the sample was centrifuged at 12,000 rpm for 10 min at 4 °C to remove unbound PEI, yielding the final PME@HPB@PEI nanozymes.

### 5.4 ABTS^+^ and DPPH· scavenging assays

#### 5.4.1 ABTS^+^ scavenging assay

A stock solution of ABTS^+^ (7.4 mM) was prepared by dissolving 3 mg of ABTS diammonium salt in 0.735 mL of distilled water. Separately, a potassium persulfate (K_2_S_2_O_8_) stock solution (2.6 mM) was prepared by dissolving 1 mg of K_2_S_2_O_8_ in 1.43 mL of distilled water. To generate the ABTS ^+^ radical cation, 0.2 mL of the ABTS stock solution was mixed with 0.2 mL of the K_2_S_2_O_8_ solution, and the mixture was incubated in the dark at room temperature for 12 h. The resulting solution was diluted 10- to 20-fold with water to achieve an absorbance of approximately 0.7 at 734 nm, yielding the working solution. For the assay, different concentrations of PME@HPB@PEI nanozymes were incubated with 1 mL of the ABTS^+^ working solution at room temperature for 15 min, after which the absorbance was measured at 734 nm using a UV-Vis spectrophotometer.

#### 5.4.2 DPPH· scavenging assay

A DPPH solution was prepared by dissolving 1 mg of DPPH in 24 mL of ethanol, followed by sonication for 5 min. This solution was then diluted with ethanol to adjust its absorbance at 519 nm to between 0.6 and 1.0, producing the working solution. Different concentrations of PME@HPB@PEI nanozymes were incubated with 1 mL of the DPPH working solution at room temperature for 30 min. The absorbance was subsequently measured at 519 nm using a UV-Vis spectrophotometer.

### 5.5 Assessment of antibacterial activity *in vitro*

The antibacterial efficacy of the materials was evaluated *in vitro* using the spread plate method following serial dilution. Briefly, *Escherichia coli* was inoculated at 1:1000 (v/v) ratio into LB broth and cultured overnight at 37 °C with shaking. Bacterial cells were then collected by centrifugation, washed, and resuspended in PBS. The bacterial suspension was incubated with different test materials for 12 h. After treatment, the samples were serially diluted, spread onto LB agar plates, and incubated at 37 °C for 12 h. The number of colony-forming units (CFU) on each plate was enumerated, and the bacterial survival rate was calculated accordingly.

### 5.6 *In vitro* evaluation of cfDNA and LPS clearance capacity

#### 5.6.1 cfDNA clearance assay

The cfDNA-binding capacity of the nanozymes was assessed using the Quant-iT™ PicoGreen™ dsDNA assay kit. A mixture containing 5 μL of cfDNA (10 μg/mL) and 5 μL of PME@HPB@PEI nanozyme suspension (5 mg/mL in PBS, pH 7.4) was thoroughly vortexed (n = 3). At predetermined time points, the supernatant was isolated by centrifugation. Then, 100 μL of the supernatant was incubated with an equal volume of PicoGreen working solution, and the fluorescence intensity was measured at excitation/emission wavelengths of 485/520 nm. The cfDNA concentration was determined based on a standard curve.

#### 5.6.2 LPS neutralization assay

The LPS adsorption capability of the nanozymes was evaluated using an LPS ELISA kit (Andy Gene, Beijing). In a typical procedure, 5 μL of LPS solution (240 ng/mL) was incubated with 5 μL of PME@HPB@PEI nanozyme suspension (5 mg/mL in PBS, pH 7.4; n = 3). At designated time points, 100 μL of supernatant was collected by centrifugation and processed according to the manufacturer's instructions. The absorbance at 450 nm was recorded, and the LPS concentration was calculated using a standard curve.

### 5.7 CLP mouse model induction

C57BL/6J mice (male, 25–30 g) were obtained from the Shanghai Laboratory Animal Research Center. Under inhalational anesthesia with 2% isoflurane, a 2 cm midline abdominal incision was made. The cecum was exteriorized, ligated 0.5 cm from the tip with a 4–0 silk suture, and punctured once with a 22-gauge needle. A small amount of fecal content was extruded, the cecum was returned, and the abdomen was closed. Sham-operated mice underwent cecal exposure without ligation or puncture. Postoperatively, all mice received subcutaneous saline (0.9%) for fluid resuscitation, analgesia, and free access to food and water.

### 5.8 Long-term biosafety assessment

Healthy C57BL/6 mice received a single injection of HPB-NPs@PME@PEI (5 mg/kg PME equivalent) or an equal volume of sterile PBS (Sham group) (n = 6 per group). At day 7 post-injection, blood samples were collected via orbital sinus puncture. Serum biochemical parameters including alanine aminotransferase (ALT), aspartate aminotransferase (AST), blood urea nitrogen (BUN), and creatinine (CRE) were measured using an automated biochemical analyzer. Serum levels of TNF-α, IL-6, IL-1β, C3a, and C5a were quantified using commercial ELISA kits (R&D Systems) according to the manufacturer's protocols.

### 5.9 LPS-induced endotoxic shock model

C57BL/6 mice received an intraperitoneal injection of LPS (10 mg/kg, from *E. coli* O111:B4, Sigma-Aldrich) to establish the endotoxic shock model. At 0.5 h post-LPS challenge, mice were intravenously administered HPB-NPs@PME@PEI (5 mg/kg PME equivalent) or an equal volume of PBS (n = 8 per group). Sham-operated mice received sterile PBS instead of LPS.

### 5.10 Human primary macrophage and neutrophil isolation and culture

Human peripheral blood mononuclear cells (PBMCs) were isolated from buffy coats of healthy donors by Ficoll-Paque PLUS (GE Healthcare) density gradient centrifugation. CD14^+^ monocytes were purified using anti-CD14 magnetic beads (Miltenyi Biotec) and differentiated into macrophages in RPMI 1640 medium supplemented with 10% FBS and 50 ng/mL GM-CSF (PeproTech) for 7 days. Primary neutrophils were isolated from peripheral blood using Polymorphprep™ (Axis-Shield) density gradient centrifugation according to the manufacturer's protocol, yielding >95% purity as verified by Wright-Giemsa staining. Cells were cultured in RPMI 1640 with 10% FBS at 37 °C in 5% CO_2_. For stimulation, primary macrophages and neutrophils were treated with LPS (100 ng/mL) in the presence or absence of HPB-NPs@PME@PEI (10 μg/mL) for 24 h. Culture supernatants were collected for cytokine analysis by ELISA, and intracellular ROS levels were measured using DCFH-DA probe (Beyotime) with fluorescence intensity quantified at Ex 488 nm/Em 525 nm.

### 5.11 Histological examination and immunohistochemistry

Lung and kidney tissues were fixed in 4% formalin, paraffin-embedded, and sectioned at 5 μm. For H&E staining, sections were deparaffinized, rehydrated, and microwaved in citric acid buffer (pH 6.0) for antigen retrieval before staining. For immunohistochemistry, sections were blocked with 10% normal goat serum containing 0.1% Triton X-100 and incubated overnight at 4 °C antibody. After washing, sections were incubated with HRP-conjugated secondary antibody (Abcam), developed with DAB, and imaged under a light microscope (Carl Zeiss).

### 5.12 Cell culture

Human alveolar epithelial (HPAEpiC) cells and human renal tubular (HK-2) cells were cultured in DMEM/F12 medium (HyClone) supplemented with 10% fetal bovine serum at 37 °C in a humidified 5% CO_2_ atmosphere.

### 5.13 Intracellular ROS quantification

Intracellular ROS levels in cells were measured using the fluorescent probe DCFH-DA (Beyotime), while intra-mitochondrial ROS levels were measured by mito-SOX probe (Dojindo). Briefly, cells were incubated with probes, washed with PBS, and analyzed (excitation: 488 and 594 nm).

### 5.14 Quantification of cell-free DNA and MPO-DNA complexes

Cell-free DNA (cfDNA) levels in serum were quantified using the Quant-iT PicoGreen dsDNA kit (Thermo Fisher) per the manufacturer's protocol. Fluorescence was measured at 480/530 nm (excitation/emission). Myeloperoxidase-DNA (MPO-DNA) complexes were detected by ELISA. Briefly, plates were coated overnight at 4 °C with an anti-MPO monoclonal antibody (Jianglon Bio). After blocking, samples were assessed with dsDNA quantification kit (Thermo Fisher).

### 5.15 Western blotting

Proteins were extracted from tissues or cells, separated by SDS-PAGE (10 and 12.5% gel, Epienzyme), and transferred to PVDF membranes. Membranes were incubated overnight at 4 °C with primary antibodies against TLR4, MyD88, p-p65, T-p65, Kim-1, iNOS (CST), and ACTIN (Abclonal).

### 5.16 Statistical analysis

Data are presented as mean ± SD. Comparisons between two groups were performed using an unpaired two-tailed Student's t-test. For multiple comparisons, one-way ANOVA followed by Tukey's post hoc test was applied. A p-value < 0.05 was considered statistically significant. All analyses were conducted using GraphPad Prism version 9.0.

## CRediT authorship contribution statement

**Yang Song:** Writing – original draft. **Xue Yu:** Writing – original draft. **Mingzhe Wu:** Writing – original draft, Formal analysis, Data curation. **Jing Liu:** Writing – original draft, Data curation, Conceptualization. **Zhifeng Wen:** Investigation, Formal analysis, Data curation. **Daosong Dong:** Investigation. **Fangdie Ye:** Writing – review & editing, Supervision, Project administration, Formal analysis. **Shijie Zhang:** Writing – review & editing, Supervision, Project administration.

## Ethics approval and consent to participate

Not applicable.

## Clinical trial number

Not applicable.

## Funding

Not applicable.

## Declaration of competing interests

The authors declare that they have no known competing financial interests or personal relationships that could have appeared to influence the work reported in this paper.

## Data Availability

Data will be made available on request.
