# Supplementary material for: Engineered hollow Prussian blue nanoparticles for synergistic anti-inflammatory therapy in sepsis
Source: Mater Today Bio. 2026 Aug 28;40:103619. doi: 10.1016/j.mtbio.2026.103619 (PMC13553568; doi:10.1016/j.mtbio.2026.103619)
Supplement: Supplementary file 1 [file mmc1.docx]

**Figure S1. TEM images of HPB (left) and PME@HPB@PEI (right) nanozymes.**

**Figure S2. Particle size and PDI stability of PME@HPB@PEI nanozymes.**

**Figure S3. PME loading efficiency of PME@HPB@PEI nanozymes.**

**Figure S4. Cy5 fluorescence biodistribution in major organs at 12，24，48 h post-injection.**

(A) Accumulation of Cy5 fluorescence in homogenized intestine tissue at different time points.

(B) Accumulation of Cy5 fluorescence in homogenized kidney tissue at different time points.

(C) Accumulation of Cy5 fluorescence in homogenized liver tissue at different time points.

(D) Accumulation of Cy5 fluorescence in homogenized lung tissue at different time points. CLP-induced septic mice received Cy5-HPB NPs@PME@PEI (5 mg/kg PME equivalent), free Cy5 dye, or PBS (n = 5). Animal tissue was perfused and organs were homogenized in RIPA buffer; Cy5 fluorescence (Ex 635 nm / Em 670 nm) was quantified. Data are mean ± SD.

**Figure S5. Long-term in vivo biosafety assessment of HPB NPs@PME@PEI.**

(A) Serum alanine aminotransferase (ALT) levels.

(B) Serum aspartate aminotransferase (AST) levels.

(C) Blood urea nitrogen (BUN) levels.

(D) Serum creatinine (CRE) levels.

(E) Serum TNF-α concentrations.

(F) Serum IL-6 concentrations.

(G) Serum IL-1β concentrations.

(H) Serum C3a concentrations.

(I) Serum C5a concentrations. C57BL/6 mice were administered HPB NPs@PME@PEI (5 mg/kg PME equivalent) or an equal volume of sterile PBS (Sham group) (n = 6 per group). At 7 days post-injection, serum samples were collected for comprehensive biochemical, inflammatory, and immunological analyses. Results are expressed as mean ± SD.

**Figure S6. HPB NPs@PME@PEI attenuates systemic inflammation, reduces cfDNA/NETosis burden, and alleviates multi-organ injury in the LPS-induced endotoxic shock model.**

Mice received sham treatment, LPS (10 mg/kg), or LPS plus HPB NPs@PME@PEI (n = 8). Serum cytokines were measured by ELISA. A. TNF-α. B. IL-6. C. IL-1β. D. IL-10. E. IFN-γ. (F-G) Serum cfDNA was quantified by PicoGreen assay; MPO-dsDNA complexes were measured by ELISA (n = 8). Serum biochemical markers of hepatic, renal, and systemic cellular injury in the LPS sepsis model (n = 8). H. ALT. I. AST. J. BUN. K. CRE. L. LDH. (M) IF analysis targeting LY6G and F4/80 in the lung of mice treated with LPS, or LPS plus HPB NPs@PME@PEI. (N) IF analysis targeting LTL and KIM-1 in the kidney of mice treated with LPS, or LPS plus HPB NPs@PME@PEI. (O) IF analysis targeting LY6G and F4/80 in the liver of mice treated with LPS, or LPS plus HPB NPs@PME@PEI. (P) WB analysis of Inos and CD86 in the mice liver. (Q) WB analysis of PAD4 in the mice liver.

**Figure S7. HPB-NPs@PME@PEI inhibits STING pathway activation in HK-2 and THP-1 cells and suppresses inflammatory responses in human primary macrophages and neutrophils.**

(A) WB analysis of HK-2 cells treated with control medium, cfDNA, cfDNA plus HPB NPs@PME, or cfDNA plus PEI alone (n = 3).

(B) WB analysis of THP-1 cells treated with control medium, cfDNA, cfDNA plus HPB NPs@PME, or cfDNA plus PEI alone (n = 3).

(C) HK-2 cells were treated with control medium, cfDNA, cfDNA plus HPB NPs@PME, or cfDNA plus PEI alone (n = 6). IFN-β in culture supernatants was measured by ELISA.

(D) THP-1 cells were treated as HK-2 cells, IFN-β in culture supernatants was measured by ELISA.

(E) Primary macrophages derived from human peripheral blood mononuclear cells were stimulated with LPS (100 ng/mL) in the presence or absence of HPB NPs@PME@PEI (n = 6), TNF-α, IL-6, and IL-1β levels in culture supernatants. DCFH-DA fluorescence intensity reflecting intracellular ROS accumulation.

(F) Primary neutrophils isolated from human peripheral blood were stimulated with LPS (100 ng/mL) in the presence or absence of HPB NPs@PME@PEI (n = 6), TNF-α, IL-6, and IL-1β levels in culture supernatants. DCFH-DA fluorescence intensity reflecting intracellular ROS accumulation.
